# Integrity of cerebellar tracts associated with the risk of bipolar disorder

**DOI:** 10.1038/s41398-022-02097-4

**Published:** 2022-08-17

**Authors:** Le Hou, Bess Yin-Hung Lam, Nichol M. L. Wong, Weicong Lu, Ruoxi Zhang, Yuping Ning, Kangguang Lin

**Affiliations:** 1 The First School of Clinical Medicine, Southern Medical University, Guangzhou, Guangdong China; 2 Department of Neurology, The Affiliated Brain Hospital of Guangzhou Medical University, Guangzhou, Guangdong Province China; 3 Department of Counselling and Psychology, Hong Kong Shue Yan University, Hong Kong, China; 4 Department of Forensic and Neurodevelopmental Sciences, Institute of Psychiatry, Psychology and Neuroscience, King’s College London, London, UK; 5 Department of Affective Disorder, The Affiliated Brain Hospital of Guangzhou Medical University, Guangzhou, Guangdong Province China; 6 School of Health and Life Sciences, University of Health and Rehabilitation Sciences, No. 17, Shandong Road, Shinan District, Qingdao, Shandong Province China

**Keywords:** Human behaviour, Neuroscience

## Abstract

This study examined the structural brain differences across individuals of different BD stages and the risks of developing bipolar disorder (BD) associated with these brain differences. A total of 221 participants who were recruited from the Guangzhou Brain Hospital and the community were categorized into four groups: NC (healthy control) (*N* = 77), high risk (HR) (*N* = 42), ultra-high risk (UHR) (*N* = 38), and bipolar disorder (BD) (*N* = 64) based on a list of criteria. Their demographics, clinical characteristics, and diffusion magnetic resonance imaging (dMRI) data were collected. ANCOVA results showed that the HR group had significantly reduced mean diffusivity (MD) (*p* = 0.043) and radial diffusivity (RD) (*p* = 0.039) of the left portico-ponto-cerebellar tracts when compared with the BD group. Moreover, logistic regression results showed that the specific diffusivity measures of cerebellar tracts (e.g., cortico-ponto-cerebellar tract), particularly the RD and MD revealed differences between groups at different BD stages after controlling for the covariates. The findings suggested that specific diffusivity (RD and MD) of cerebellar tracts (e.g., cortico-ponto-cerebellar tract) revealed differences between groups at different BD stages which is helpful in detecting the trajectory changes in BD syndromes in the early stages of BD, particularly when the BD syndromes start from HR stage.

## Introduction

Bipolar disorder (BD) is a highly heritable disabling mental illness (higher than 70%) [1], affecting the patients and more broadly their caregivers as well as the healthcare system [2, 3]. BD afflicts approximately 1–2% of the general population [4], characterized by depression and hypo/manic episodes. Extant evidence suggests that for many patients there are two stages—namely high-risk (HR)and ultra-high-risk (UHR)—before the official onset of the disease. It is of particular interest to investigate the BD-related symptoms in bipolar offspring as they have been repeatedly found to be related to an elevated risk of developing future full-blown BD [5]. Hence, from the perspective of diffusion magnetic resonance imaging (dMRI), the present study would examine the structural brain differences across individuals of different BD stages (i.e. HR and UHR) and the risks associated with these brain differences.

## Early stages of BD

In recent decades, more light has been shed on the trajectory of BD and the identification of early-risk syndromes that precede full-blown BD [6–8]. Specifically, these findings suggested that the early symptoms often start off non-specifically during childhood (e.g., anxiety) and then further develop during early adolescence and later as subclinical depression and/or hypomania that reaches the official BD criteria [6–8]. Accumulating evidence has supported that there are early stages preceding the full-blown BD—HR and UHR. According to Scott et al. [9] and Frank et al. [10], the HR stage is characterized by the phase of biological vulnerability (i.e., genetic risk) with no or mild, non-specific symptoms while UHR individuals manifest subthreshold syndromes, alterations in cortical (and subcortical) volumes, and deficits in cognitive function. These findings regarding early BD syndromes are crucial for the prevention and intervention of BD.

## MRI findings on BD and early BD stages

Given that it is best to prevent and intervene at earlier stages [11], there is an emerging need to investigate the predictive factors particularly the neuroanatomical underpinnings of early BD syndromes. Although there is increasing research pertaining to the behavioral outcomes [12, 13], previous brain studies are still focusing on BD while HR and UHR individuals are often overlooked [14, 15]. For instance, consistent findings consistently found abnormal resting-state functional connectivity patterns among the corticolimbic and cerebellar networks among BD patients [16–18]. Although some researchers also found changes in the corticolimbic, striatal and cerebellar resting-state functional connectivity patterns among unaffected relatives of BD patients [19], prior findings were limited, inconsistent, and negative [20, 21].

Among the few studies regarding the neuroimaging underpinnings in HR and UHR, Lin et al. [14] found distinct patterns of atypical resting-state signals and functional connectivity that predicted cognitive functioning in the offspring of parents with BD in the HR and UHR stages. Specifically, it was found that the inferior frontal cortex, the striatum, cerebellum, and the hippocampus, as well as the lateral prefrontal cortex, were associated with different neuropsychological functions including the speed of processing, mnemonic, processing speed, and executive performance. In particular, processing speed, attention, and verbal learning/memory were positively correlated with the functional connectivity between the left hippocampus and cerebellum in the UHR offspring. Moreover, even less is known about the structural connectivity across different stages of BD when compared with functional connectivity findings. One recent study by Bora and colleagues found that reduced regional connectivity in the right occipito-parietal areas and cerebellum was evident in clinical high-risk and those with no such history [22]. Furthermore, decreased interregional connectivity between nodes in the right and left prefrontal regions, nodes in the right prefrontal lobe and right temporal lobe, and nodes in the left occipital area and left cerebellum were evident in clinical high-risk individuals. These findings lead to the speculation that structural brain connectivity is linked to vulnerability to BD and predictive of the emergence of manic symptoms. For instance, a review study indicated a significant role of the cerebellum in BD [23]. Specifically, 12 studies investigated structural and functional neuroimaging of the cerebellum in BD and showed that the cerebellum plays a role in modulating emotional processing. These findings provide strong support for the clinical relevance of cerebellar-limbic connections and indication of the cerebellum as an “emotional pacemaker”. Another previous study also investigated the cerebellar size in BD patients [24]. It was found that the V3 area was significantly smaller in multiple-episode patients than in first-episode patients and healthy volunteers. These results suggested that cerebellar vermal atrophy was found in patients with bipolar disorder who had multiple affective episodes. Along with the mounting evidence suggesting that the cerebellum is a key region of interest (ROI) contributing to various cognitive functions (e.g., executive control and sensorimotor processing) [25–34], the present study aimed to study the cerebellar tracts in HR, UHR, and BD against healthy controls.

## Study aims and hypotheses

Specifically, the bilateral cortico-ponto-cerebellar tracts, the superior cerebellar tracts, and the inferior cerebellar tracts, defined by tractography-based atlas [35], were our tracts of interest. With the dMRI approach, the present study aimed to investigate the differences in the fractional anisotropy (FA), mean diffusivity (MD), axial diffusivity (AD), and radial diffusivity (RD) of each tract of interest between healthy controls and three different stages of bipolar disorder (HR, UHR, and BD). For tracts that showed significant group differences (*p* < 0.05), it was hypothesized that the specific diffusivity measures of these tracts revealed differences between groups at different BD stages, even after controlling for their age, gender, anxiety, depression, and psychotic symptoms.

## Materials and methods

### Participants

The present study was a part of the Recognition and Early intervention on Prodromal Bipolar Disorder (REI-PBD) project [13]. The data of this study were collected from March 2013 to December 2017. This study was approved by the Institutional Review Board of The Affiliated Brain Hospital of Guangzhou Medical University. A total of 221 (111 males, 110 females) participants were recruited from the Hospital and volunteers from the community. All participants (if participants were aged 18 years and above) or their guardians (if participants were aged under 18 years) gave written informed consent before their participation in this study. Based on the criteria listed below, the participants between 8 and 28 years old (mean age = 17.95 years, SD = 4.76 years) were categorized into four groups: NC (*N* = 77), high risk (HR) (*N* = 42), ultra-high risk (UHR) (*N* = 38) and BD (*N* = 64).

NC were individuals with no BD family history while they had never been diagnosed with any DSM-IV-TR Axis I disorder. The diagnosis of BD was based on the Structured Clinical Interview for DSM-IV-TR Axis I Disorders (SCID-I).

The criteria for the HR and UHR were adapted from Lin et al. [13]. The inclusion criteria for HR offspring were as follows: (i) offspring of at least one biological parent with bipolar disorder; (ii) age between 8 and 28 years old; and (iii) a lack of precursor syndromes (see below) and no diagnosis with any DSM-IV-TR Axis I disorder. Participants who met any of the following conditions were excluded: any DSM-IV-TR Axis I disorders, drug or alcohol abuse, pregnancy, hypothyroidism, hyperthyroidism, or craniocerebral trauma.

The criteria for the UHR offspring were: (i) offspring of at least one biological parent with bipolar disorder; (ii) age between 8 and 28 years old; and (iii) at least one of the following syndromes must be met: (1) two or more hypomania symptoms have been present at least 4 days but not meeting DSM-IV hypomania episode criteria; (2) two or more major depressive symptoms have been present at least 1 week but not meeting DSM-IV major depressive episode criteria; (iii) one or more attenuated psychotic symptoms present and last at least 10 min for each manifestation and 2–7 manifestations per week for at least 3 months. The attenuated psychotic symptoms are as follows: ideas of reference, odd ideas, odd beliefs, unusual perceptual experiences, bizarre thoughts or speech, grandiosity, suspicious ideas, paranoid ideas, odd mannerisms, hallucinations, disorganized/catatonic behaviors; (iii) two or more DSM-IV-TR defined hyperactivity and impulsivity symptoms/signs were observable by teachers, peers, and/or parents.

### Measures

Anxiety, depression, and psychotic symptoms of all participants were measured by 17-item Hamilton Depression Rating Scale (HAMD) [36], the HAMA [37], and the 18-item Brief Psychiatric Rating Scale [38], respectively. All these measures received good reliabilities.

### MRI data acquisition and preprocessing

The diffusion MRI (dMRI) data from the participants were collected on a Philips Achieva X-series 3.0 Tesla scanner with an 8-channels SENSE head coilin the Affiliated Brain Hospital of Guangzhou Medical University. The dMRI data were acquired with a non-diffusion-weighted image (*b*_0_) with *b*-value = 1000 s/mm^2^ using the following parameters: TR = 10,100 ms, TE = 90 ms, FOV = 256 × 256 mm^2^, voxel size = 2 × 2 × 2 mm^3^. The number of diffusion sampling directions for the *b*-value was 32.

The dMRI data acquired were corrected for eddy current distortion by registering each volume of the participant’s dMRI data to their *b*_0_ image using *eddy_correct* [39] in FSL [40] (https://fsl.fmrib.ox.ac.uk/fsl/fslwiki/). The data were inspected for motion artifacts and no data were subsequently removed. The diffusion tensor model was fitted to the data at every voxel to obtain fractional anisotropy (FA), mean diffusivity (MD), axial diffusivity (AD), and radial diffusivity (RD) map for each participant. All of the FA maps were aligned to the default FMRIB58_FA template and were transformed to the Montreal Neurological Institute (MNI) standard space via a nonlinear registration. All participants’ FA, MD, AD, and RD maps were subsequently transformed to the MNI standard space [41].

**Fig. 1:**
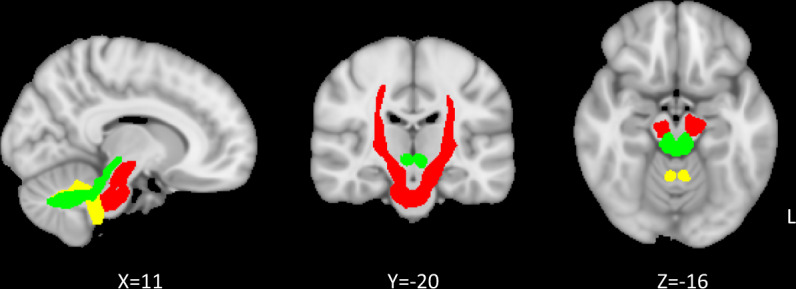
Tracts of interest. The bilateral cortico-ponto-cerebellar tracts, the superior cerebellar tracts, and the inferior cerebellar tracts, defined by tractography-based atlas, are presented in red, green, and yellow respectively. L = left.

Based on our a priori interest, the bilateral cortico-ponto-cerebellar tracts, the superior cerebellar tracts, and the inferior cerebellar tracts, defined by the tractography-based atlas [42], were our tracts of interest (Fig. 1), and the mean FA, MD, AD, RD within each tract of each participant was retrieved for analyses.

### Statistical analyses

One-way Analysis of variance (ANOVA) and Chi-square analyses were performed to investigate the group differences in demographics and clinical characteristics. For FA, MD, AD, and RD of each tract, one-way Analysis of Covariance (ANCOVA) was performed using *ezANOVA* from the R package*ez* (https://cran.r-project.org/web/packages/ez), controlling for age, gender, HAMA, HAMD, and BPRS. The statistical threshold for *p* values was defined at 0.05. To correct for the number of tracts and the diffusivity measures, both their uncorrected *p* and Bonferroni-corrected *p*_corrected_ values would be reported. Post-hoc *t* statistics, corrected for the number of pairs of groups, would be performed on the significant tracts.

As MD and RD measures of the left cortico-ponto-cerebellar tract showed significant group differences (*p*_corrected_ < 0.05), we further investigated the ordinal logistic regression models with MD or RD of the left cortico-ponto-cerebellar tract (in 10^−4^ unit) as a statistical predictor for classifying different stages for the bipolar disorder (NC, HR, UHR, and BD) using *polr* from the R package *MASS* (https://cran.r-project.org/web/packages/MASS), after controlling for age, gender, HAMA, HAMD, and BPRS. Binary logistic regression analyses would also be conducted between any pairs of groups using MD or RD of the left cortico-ponto-cerebellar tract (in 10^−4^ unit) as a statistical predictor after controlling for age, gender, HAMA, HAMD, and BPRS. The significance of the logistic regression models was evaluated based on the *t* statistics. In the binary logistic regression analyses, *p*_corrected_ values would be reported, and corrected for the number of pairs of groups. All the analyses were performed in R (https://www.r-project.org/).

## Results

### Group differences in demographics, clinical characteristics, and cerebellar tracts

#### Demographics and clinical characteristics

The mean and standard deviation of these characteristics for each group (NC, HR, UHR, and BD) were reported in Table 1. Specifically, it was revealed that the four groups differed in terms of age, gender ratio, scores in HAMA, HAMD, and BPRS (*p*s < 0.05). Therefore, they were all included as covariates in the subsequent analyses so as to minimize any possible confounding effects.

**Table 1:** Demographics and clinical characteristics for each group (normal healthy control, HR, URH, and BD) with group difference statistics.

Group	High risk (HR) (*N* = 42)	Ultra-high risk (UHR) (*N* = 38)	Bipolar disorder (BD) (*N* = 64)	Healthy control (NC) (*N* = 77)	Total sample (*N* = 221)	Chi-square/*F*-statistics
Gender	17 (40.5%) females and 25 males (59.5%)	20 females (52.6%) and 18 males (47.4%)	41 females (64.1%) and 23 males (35.9%)	32 females (41.6%) and 45 males (58.4%)	110 females (49.8%) and 111 males (50.2%)	*X*^2^(3) = 8.88, *p* = 0.031*
Age (SD) in years	17.95 (4.76)	16.79 (5.43)	20.73 (3.60)	15.36 (3.25)	17.66 (4.61)	*F*(3217) = 20.764, *p* < 0.001***
HAMD	0.44 (0.90)	7.08 (9.27)	2.98 (3.76)	0.30 (0.80)	2.26 (4.99)	*F*(3217) = 23.09, *p* < 0.001***
HAMA	0.49 (0.93)	5.51 (7.97)	1.79 (2.12)	0.38 (1.13)	1.68 (4.00)	*F*(3217) = 19.14, *p* < 0.001***
BPRS	17.83 (2.92)	22.81 (5.34)	20.07 (3.19)	18.04 (2.27)	19.40 (3.79)	*F*(3217) = 20.77, *p* < 0.001***

*HAMD* Hamilton Rating Scale for Depression, *HAMA* Hamilton Rating Scale for Anxiety, *BPRS* Brief Psychiatric Rating Scale.

*p < 0.05; ***p < 0.001.

#### Cerebellar tracts

The mean and standard deviation of the diffusivity measures for each group (NC, HR, URH, and BD) were reported in Table 2.

**Table 2:** Diffusivity measures for each group (normal healthy control, HR, URH, and BD).

Group	High risk (HR) (*N* = 42)		Ultra-high risk (UHR) (*N* = 38)		Bipolar disorder (BD) (*N* = 64)		Healthy control (NC) (*N* = 77)	
	Mean	SD	Mean	SD	Mean	SD	Mean	SD
*FA*								
Left cortico-ponto-cerebellar tract	0.5079	0.01415	0.5061	0.01508	0.5042	0.01448	0.5056	0.01295
Right cortico-ponto-cerebellar tract	0.5345	0.01394	0.5318	0.01612	0.5308	0.01621	0.5304	0.01219
Left superior cerebellar tract	0.3631	0.01318	0.3591	0.01412	0.3579	0.01351	0.3594	0.01045
Right superior cerebellar tract	0.3686	0.01203	0.3665	0.01517	0.3664	0.01334	0.3660	0.00942
Left inferior cerebellar tract	0.3367	0.01251	0.3332	0.01608	0.3317	0.01372	0.3312	0.01075
Right inferior cerebellar tract	0.3424	0.01349	0.3396	0.01627	0.3391	0.01352	0.3379	0.01069
*MD*								
Left cortico-ponto-cerebellar tract	0.0008	0.00003	0.0008	0.00002	0.0008	0.00003	0.0008	0.00002
Right cortico-ponto-cerebellar tract	0.0007	0.00002	0.0008	0.00002	0.0008	0.00002	0.0008	0.00002
Left superior cerebellar tract	0.0012	0.0001	0.0012	0.00007	0.0012	0.00007	0.0012	0.00006
Right superior cerebellar tract	0.0011	0.00008	0.0011	0.00006	0.0011	0.00006	0.0011	0.00005
Left inferior cerebellar tract	0.0009	0.00004	0.0009	0.00003	0.0010	0.00004	0.0010	0.00004
Right inferior cerebellar tract	0.0009	0.00004	0.0009	0.00003	0.0009	0.00004	0.0009	0.00004
*AD*								
Left cortico-ponto-cerebellar tract	0.0013	0.00004	0.0013	0.00003	0.0013	0.00003	0.0013	0.00002
Right cortico-ponto-cerebellar tract	0.0012	0.00003	0.0012	0.00003	0.0012	0.00003	0.0013	0.00002
Left superior cerebellar tract	0.0015	0.00011	0.0015	0.00008	0.0016	0.00007	0.0016	0.00006
Right superior cerebellar tract	0.0015	0.00009	0.0015	0.00007	0.0015	0.00006	0.0015	0.00006
Left inferior cerebellar tract	0.0013	0.00005	0.0013	0.00004	0.0013	0.00004	0.0013	0.00004
Right inferior cerebellar tract	0.0013	0.00005	0.0013	0.00004	0.0013	0.00004	0.0013	0.00004
*RD*								
Left cortico-ponto-cerebellar tract	0.0005	0.00003	0.0005	0.00002	0.0005	0.00003	0.0005	0.00002
Right cortico-ponto-cerebellar tract	0.0005	0.00002	0.0005	0.00002	0.0005	0.00002	0.0005	0.00002
Left superior cerebellar tract	0.0010	0.00009	0.0010	0.00006	0.0010	0.00007	0.0010	0.00005
Right superior cerebellar tract	0.0009	0.00007	0.0009	0.00006	0.0009	0.00006	0.0009	0.00005
Left inferior cerebellar tract	0.0008	0.00004	0.0008	0.00003	0.0008	0.00004	0.0008	0.00003
Right inferior cerebellar tract	0.0008	0.00004	0.0008	0.00003	0.0008	0.00004	0.0008	0.00003

*FA* fractional anisotropy, *MD* mean diffusivity, *AD* axial diffusivity, *RD* radial diffusivity.

With regard to the FA of the cerebellar tracts, significant group differences were observed in the left inferior cerebellar (*F*(3211) = 3.53, *p* = 0.016) and left superior cerebellar tracts (*F*(3211) = 4.33, *p* = 0.005) but they did not survive the correction for multiple comparisons (*p*_corrected_ > 0.05).

For MD, group differences were observed in the bilateral cortico-ponto-cerebellar tracts (left: F(3211) = 6.61, *p* < 0.001; right: *F*(3211) = 3.56, *p* = 0.015) and ﻿left superior cerebellar tracts (*F*(3211) = 2.61, *p* = 0.052 (marginal significant)), and only the left cortico-ponto-cerebellar tract remained significant after correction for multiple comparisons (*p*_corrected_ = 0.003).

For AD, group difference was also observed in the left cortico-ponto-cerebellar tract (*F*(3211) = 3.98, *p* = 0.009) but it did not survive the correction for multiple comparisons (*p*_corrected_ = 0.106).

For RD, bilateral cortico-ponto-cerebellar (left: *F*(3211) = 6.51, *p* < 0.001; right: *F*(3311) = 3.83, *p* = 0.011) and bilateral superior cerebellar tracts (left: *F*(3211) = 3.02, *p* = 0.031; right: *F*(3311) = 2.73, *p* = 0.045), and only the left cortico-ponto-cerebellar tract remained significant after correction for multiple comparisons (*p*_corrected_ = 0.004).

**Fig. 2:**
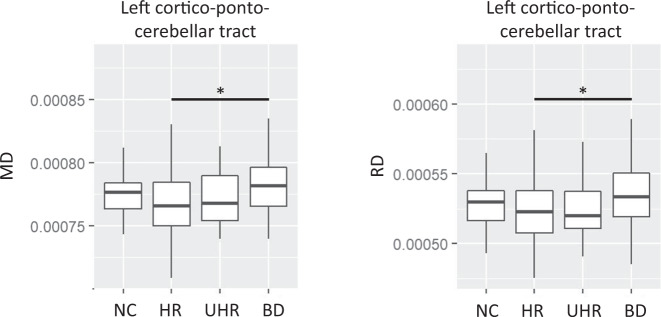
Diffusivity of left cortico-ponto-cerebellar tract. Left cortico-ponto-cerebellar tract showed significant group differences in mean diffusivity (MD) and radial diffusivity (RD) between people of different stages of bipolar disorder (BD). Post-hoc comparisons revealed that BD group had significantly increased MD (*p*_corrected_ = 0.043) and RD (*p*_corrected_ = 0.039) in the tract when compared with the high-risk (HR) group. NC normal healthy controls, UHR ultra-high risk. *p_corrected_ < 0.05.

In the post-hoc analyses, BD group had significantly increased MD (*t* = 2.75, *p*_corrected_ = 0.043) and RD (*t* = 2.78, *p*_corrected_ = 0.039) in the left cortico-ponto-cerebellar tract when compared with the HR group. No other significant differences were found (Fig. 2).

#### Cerebellar tracts predicting the risk for bipolar disorder

Based on current findings regarding the group differences in tracts, two ordinal logistic regression analyses were performed on including MD or RD of the left cortico-ponto-cerebellar tract (in 10^−4^ unit) as a statistical predictor, in addition to age, gender, HAMA, HAMD, and BPRS, to investigate their predictions on classifying people into healthy or different stages for bipolar disorder. In separate ordinal logistic regression models, MD (OR = 1.27, *p* = 0.167) or RD (OR = 1.37, *p* = 0.082) of the left cortico-ponto-cerebellar tract were not significant.

In the binary logistic regression models, MD of the left cortico-ponto-cerebellar tract (in 10^−4^ unit) statistically predicted the classification of people between NC and BD (OR = 121.03, *p*_corrected_ = 0.030), between HR and BD (OR = 182.73, *p*_corrected_ = 0.007), and marginally significant between UHR and BD (OR = 40.02, *p*_corrected_ = 0.051) after correction for multiple comparisons. Similarly, RD of the left cortico-ponto-cerebellar tract (in 10^−4^ unit) statistically predicted the classification of people between NC and BD (OR = 78.58, *p*_corrected_ = 0.042), between HR and BD (OR = 184.32, *p*_corrected_ = 0.009), and between UHR and BD (OR = 43.99, *p*_corrected_ = 0.033).

## Discussion

In the present study, the specific diffusivity measures of cerebellar tracts were investigated and compared across three different stages of BD (HR, UHR, and BD) and healthy controls. In addition, the risks for developing BD associated with these brain differences were examined. The key findings, which are consistent with the hypothesis and prior findings [26–28, 30, 31], showed significant group differences (HR vs. BD) in terms of the specific diffusivity measures (MD and RD) of cerebellar tracts. Furthermore, the specific diffusivity measures of cerebellar tracts (MD and RD) revealed differences between groups at different BD stages, even after controlling for their age, gender, anxiety, depression, and psychotic symptoms. Taken all these results together, specific diffusivity (MD and RD) but not FA revealed differences between groups at different BD stages which consistently support the role of the cerebellum in the cognition and affective state and BD [25–34]. More importantly, the current findings are helpful in detecting the trajectory changes in BD syndromes in the early stages of BD.

With the present data, although group differences were observed in the specific diffusivity measures of cerebellar tracts (the MD and RD of the cortico-ponto-cerebellar tract), the only significant post-hoc differences were found between the HR and BD groups. Specifically, it was observed that the BD group had significantly reduced MD and RD of the leftcortico-ponto-cerebellar tracts when compared with the HR group. These findings were consistent with prior literature suggesting that a reduction in cerebellar white matter and correlates were coupled with worsened BD syndromes [23, 24, 42]. However, no significant group differences were observed pertaining to the FA of any cerebellar tracts which did not support the hypothesis. It is possible that FA of cerebellar tracts, which is a summary measure of microstructural integrity, may not be as sensitive as MD and RD to detect the trajectory changes in BD syndromes in the early stages of BD.

Logistic regression results showed that the specific diffusivity measures of cerebellar tracts, particularly the RD and MD but not FA revealed differences between groups at different BD stages, even after controlling for their age, gender, anxiety, depression, and psychotic symptoms. This finding is consistent with the hypothesis. Specifically, when compared HR and UHR with BD, respectively, MD and RD of the left cortico-ponto-cerebellar tract significantly increased the predictability of BD risk. When comparing BD and healthy controls, MD and RD of the left cortico-ponto-cerebellar tract also significantly increased the model prediction of BD risk. With the current data, consistent findings regarding the cortico-ponto-cerebellar tract diffusivity measures suggest that these revealed differences between groups at different BD stages [23, 24, 42]. However, it was found that cerebellar tracts did not differ between HR and UHR in the present study which did not support the hypothesis. This might be because individuals at these two BD stages were not affected yet, which is reflected in their cerebellar tracts. Taken together, specific diffusivity (RD and MD) but not FA revealed differences between groups at different BD stages which is helpful in detecting the trajectory changes in BD syndromes in the early stages of BD, particularly when the BD syndromes start from the HR stage.

### Limitations

Despite the important implications, the present study has a number of limitations. First, it was a cross-sectional study that cannot establish the causal relationship between cerebellar tracts and the risk of developing BD. However, this sets a foundation for future studies to further delineate the causality. Longitudinal studies are needed for investigating whether the structural brain differences are the biomarkers and predictors of developing BD. Secondly, some variables such as social-cognitive functions which might be important to the development of BD were not measured. Future studies might consider including those variables in understanding the development of BD. Last but not least, the current sample size might be small for the logistic regression but the current findings with four groups of individuals at four different BD stages are important for supporting future studies with larger sample sizes to investigate the causal relationship between cerebellar correlates and BD.

## Conclusion

The present study examined the structural brain differences across healthy individuals and individuals of different BD stages (high risk, ultra-high risk, and BD) and the risks for developing BD associated with these brain differences. The current findings suggested that specific diffusivity (RD and MD) of cerebellar tracts (e.g., cortico-ponto-cerebellar tract) revealed differences between groups at different BD stages which is helpful in detecting the trajectory changes in BD syndromes in the early stages of BD, particularly when the BD syndromes start from HR stage. These findings help us gain a better understanding of the neuroanatomical underpinnings of different stages of BD which set the foundation for the development of prevention and early intervention of BD.

## Data Availability

Data are available upon request.
